# A comparative study of food safety knowledge among mobile food vendors and canteen food handlers in markets in Kano Metropolis, Northwest Nigeria

**DOI:** 10.11604/pamj.2024.48.131.36934

**Published:** 2024-07-24

**Authors:** Usman Muhammad Ibrahim, Zahrau Zubairu, Surayya Murtala Sunusi, Suraj Musa Inuwa, Ado Shehu, Sagir Magaji, Aliyu Ahmed Sadiq, Shamsuddeen Abdullahi, Yusuf Karkarna Mustapha, Sunday Audu, Jamilu Aliyu Bawa, Abba Ahmed Danzomo, Usman Bashir, Abubakar Mohammed Jibo, Muhammad Lawan Umar

**Affiliations:** 1 Department of Community Medicine, Aminu Kano Teaching Hospital, Kano, Nigeria,; 2 Department of Nursing Science, Aminu Kano Teaching Hospital, Kano, Nigeria,; 3 Department of Nursing Science, Federal Medical Center Birnin Kudu, Birnin Kudu, Nigeria,; 4 Department of Microbiology, Bayero University Kano, Kano, Nigeria,; 5 University of Essex, Colchester, United Kingdom,; 6 World Health Organization, Jigawa State Field Office, Jigawa State, Nigeria,; 7 Department of Community Medicine, Bayero University Kano, Aminu Kano Teaching Hospital, Kano, Nigeria

**Keywords:** Food safety and hygiene, knowledge, practice, vendors, Kano, Nigeria

## Abstract

**Introduction:**

foodborne diseases are an emerging public health challenge due to the global increase in vended food. This study aimed to compare food safety knowledge among mobile and canteen food handlers in Kano metropolis.

**Methods:**

a comparative cross-sectional design was used to study 310 mobile food vendors and 310 canteen food handlers selected using a multi-stage sampling technique. Data were collected by interviewer-administered questionnaire and analyzed at univariate, bivariate, and multivariate levels using SPSS version 20 at 5% α level of significance.

**Results:**

the mean ages (±SD) of mobile and canteen food vendors were 24.6±9.1 and 32.1±10.3 years, respectively. Majority of them were females (66.2% mobile and 61.5% canteen food vendors). Good knowledge of food safety and hygiene was found among 22 (7.2%) and 67 (23.3%), mobile and canteen food vendors, respectively. Mobile food vendors who worked for less than 35 hours per week were 70% less likely to have good knowledge of food safety [AOR=0.3, 95%CI=0.2-0.6, p<0.001] relative to those who worked for ≥35 hours per week. Among canteen food vendors, marital status [AOR=1.7, 95%CI=1.2-1.3, p=0.002], hours of food vending per week [AOR=2.7, 95%CI=1.6-4.3, p<0.001], and job description [AOR=0.5, 95%CI=0.3-0.9, p=0.008], were independent predictors of food safety knowledge.

**Conclusion:**

knowledge of food safety and hygiene was found to be suboptimal among both canteen food vendors and mobile food vendors. The government should ensure regular training and supervision of food vendors for compliance with food safety guidelines.

## Introduction

Food handlers play a significant role in ensuring food safety throughout the chain of production, processing, storage, and preparation [1,2]. A significant burden of food-borne disease outbreaks is due to contamination by food handlers [2-5]. Poor handling of food and non-compliance with hygienic measures enable disease-causing organisms to come into contact with food, survive and multiply in adequate numbers to result in morbidity, mortality and significant socio-economic burden [1-5]. Poor personal hygiene and environmental conditions facilitate the transmission of foodborne diseases [1-5]. Sale and consumption of vended foods are increasing globally due to urbanization [4-9]. In developing countries including Nigeria, there is a noticeable increase in the number of street food vendors as a result of dwindling economy and unemployment [6].

According to the Food and Agricultural Organization, about 2.5 billion people consume street vended food daily with many of the ingredients required for the preparation sourced in small quantities from local suppliers and therefore prone to contamination and resultant infection [10]. Epidemiological studies have shown a significantly high prevalence of food-borne disease of 10% to 15% and in the late 1990s, more reliable data from the US suggested that this figure may be up to 25%. Similarly, comparable studies in developing countries that identified the burden of food-borne diseases are lacking and the assumption is such that food-borne disease prevalence is higher in these countries and the health and economic impact more pronounced [11]. Foodborne diseases not only adversely affect people´s health and well-being, but may also result in negative social and economic consequences for individuals, families, communities, businesses and countries [12-20]. These diseases also impose a substantial burden on healthcare systems, trade and tourism, markedly reduce economic productivity and threaten livelihood [12].

In Kano Nigeria, the retail food sector is progressively increasing with a significant number of mobile and canteen food vendors serving a good number of people within Kano and those coming to Kano for commercial activities. The two categories of food vendors are involved in food handling, provides services to consumers on daily basis. They are likely to differ in terms of their knowledge of food safety and hygiene [5,13]. In addition, food sold in the open by mobile food vendors are unlikely to be prepared and sold under hygienic conditions perhaps due to improper conditions related to food preparation, storage, and waste disposal [12-16]. They are also unlikely to be supervised by the designated regulatory agencies since they might not be licensed and often predominantly prepare food at home but served and consumed on the street [17]. This highlights the enormous responsibility on the food and agro-allied industries, food safety regulatory agencies and other stakeholders in the food supply chain to ensure that the public consumes safe, wholesome and nutritious food [4].

However, the food safety and hygiene legislations among food vendors are unlikely to be well implemented in northern Nigeria including Kano, and documented evidence on comparative study of food safety and hygiene knowledge among vendors in Kano is generally lacking. There is therefore an urgent need to strengthen the existing food safety system at each enterprise level at all levels to develop a safe and reliable food supply chain [13]. This study was done to compare the knowledge of food safety and hygiene among canteen and mobile food vendors in Kano metropolis, Nigeria. Findings from the study will provide evidence to policy makers to enforce the existing food safety and hygiene policy and legislation in Kano.

## Methods

**Study area:** Kano state is one of the states in Northwestern Nigeria. The state has forty-four (44) Local Government Areas (LGAs). The capital of Kano State is Kano city, which has eight metropolitan LGAs consisting of Dala, Fagge, Gwale, Kano Municipal, Kumbotso, Nasarawa, Tarauni and Ungogo. The metropolitan LGAs serve as the commercial centers of the state and other neighboring states of the country within which all the major markets in Kano State are distributed. There are about 40 major markets of different varieties of commodities spread across the metropolis, with most of them operating daily between the hours of 9am to 10pm [19,20]. There are many mobile and canteen food vendors in the markets. They serve as the major source of food supply with varying degrees of hygiene for those involved in business activities. The certification for food vending is the responsibility of the occupational health unit of Kano State Ministry of Health. There are currently over 5000 registered canteens in the state and more than 3800 are located within the metropolis. There is no register in the state for mobile food vendors. Data was collected between 10^th^ November 2018 and 11^th^ April, 2019.

**Study population:** the licensed and non-licensed mobile food vendors and canteen food handlers in Kano metropolis. Those who have been in the cooked food business for six months or more qualified for inclusion while any identified person involved in administrative activities among the two groups of study participants and those below the age of 18 years were excluded.

**Sample size estimation:** the sample size was calculated using the formula for comparing two proportions [21].

$$n=\frac{{\left(Z_{\alpha }+Z_{1-\beta }\right)}^{2}\left[\left(P_{1}\left(1-P_{1}\right)+P_{2}\left(1-P_{2}\right)\right)\right]}{{\left(P_{1}-P_{2}\right)}^{2}}$$

With Z_α_= 1.96, Z_1-β_= the probability of type II error (β) of power at 80%= 0.84 P_1_= proportion of mobile food vendors in Kano with good food safety practices= 93.2%= 0.932 [22]. P_2_=proportion of canteen food handlers in Sokoto with good food safety practices 86.3%= 0.863 [23]. Using a non-response rate of 10% from the previous study [3] n=310 per group were studied.

**Study design:** comparative cross-sectional study design was used.

**Sampling technique:** multistage sampling technique involving four stages was used to study the eligible food vendors.

***Stage one: selection of major markets:*** a list of all the major markets (markets operating from Monday to Sunday) in Kano metropolis was obtained [20]. Major markets were selected because of the possibility of having large number of mobile and canteen food vendors with significant patronage by people in the market. Using the list, ten (10) markets were selected by simple random sampling using balloting technique from the list of 40 major markets.

***Stage two: selection of canteens in the selected major markets:*** a census was conducted in the selected major markets to obtain the total lists of canteens and stalls. This was conducted by mapping all the streets in the markets, using the traditional names assigned to the streets, and numbers were allocated to all the canteens in the mapped streets from which the total number of 617 canteens was obtained. The canteens to be studied (310) were selected by simple random sampling/balloting technique using the numbers assigned to the canteens during mapping and numbering.

***Stage three: selection of canteen food handlers:*** in each of the selected canteens, the list of all the food vendors eligible for the study was generated and served as the sampling frame for the canteen. One food vendor was selected using simple random sampling technique by balloting and interviewed in each of the selected canteens.

***Stage four: selection of mobile food vendors:*** the mobile food vendors were noted to be distributed in clusters across the selected major markets. A census was conducted to obtain the total number of clusters in each of the selected markets and the average number of mobile food vendors in each cluster. Numbers were assigned to the identified clusters of mobile food vendors across all the selected markets. The sample size of 310 was proportionately allocated based on the total number of food vendors in each cluster and eligible respondents were randomly selected by balloting. If the number of mobile food vendors in the selected cluster did not meet up the proportionately allocated numbers, the next available clusters were considered in a similar pattern until the allocated sample size was obtained.

**Instrument and method of data collection:** pre-tested structured interviewer-administered adapted questionnaire [9,18,22,23] was used to collect data from the respondents. The questions were based on the recommended WHO concept of five keys to safer foods. Twenty research assistants who were Community Health Extension Workers were recruited and trained for this study (two for each of the markets selected). The training sessions lasted for three days and covered an overview of the research, how to obtain informed consent, communication skills, how to administer and fill questionnaires appropriately, advocacy and community entry, and mapping and numbering of street food vendors. Thirty-one (31) mobile and canteen food vendors respectively were studied in pre-testing the data collection tool outside Kano metropolis. The questionnaires used for pretesting were analysed to assess the suitability of the tool, time needed to administer, social acceptability, and appropriate reviews were made to standardize the data collection process before conducting the study.

**Data management:** data were analyzed using SPSS Statistical software version 20.0 after appropriate cleaning. Twenty-four (24) questions were used to assess knowledge of food safety and hygiene. Each correct spontaneous response was given one point; assisted or probed awareness was scored half a point; wrong response was awarded zero point and the total scores were summed up [24,25]. Total food safety knowledge score of < 9 was considered as poor knowledge of food safety and hygiene while scores of 9-13 and >13 were considered as fair and good food safety knowledge, respectively [22,23,25]. The outcome variable is food safety and hygiene knowledge while the independent variables are age, sex, and duration in food vending business among others. The Chi-squared test was used for comparison of proportions at ≤ 5% α- level of significance. Factors significantly associated with food safety and hygiene knowledge ≤ 0.1 at bivariate level were entered into a logistic regression model to adjust for confounding [24,25].

**Ethical considerations:** Health Research Ethics Committee of the Kano State Ministry of Health (with approval number: MOH/OFF/797/T1/733) and Aminu Kano Teaching Hospital (with approval number: NHREC/21/08/2008/AKTH/EC/2260) provided ethical clearance for the study. Written informed consent was obtained from all the respondents selected for participation in this study using the consent forms. All provisions of Helsinki Declaration were adhered to during the study.

## Results

Out of the total of the 310 questionnaires distributed, each to the mobile food vendors and canteen food vendors, 305 and 288 questionnaires were retrieved giving response rates of 98.4% and 92.9%, respectively. Table 1 shows that the mean ages (±SD) of street food vendors and canteen food handlers were 24.6±9.1 and 32.1±10.3 years, respectively. Female food vendors were the majority among the street mobile (66.2%) and canteen 177 (61.5%) food handlers. One hundred and ninety-five (67.7%) of the canteen food handlers had ≥5 years´ work experience in food vending while 162 (53.0%) of mobile food vendors had < 5 years´ work experience. Only 28 (9.0%) mobile food vendors and 45 (15.6%) canteen food vendors learnt food preparation from catering school. One hundred and seven (35.0%) of mobile food vendors and 103 (35.8%) canteen food vendors gave a history of vaccination against either hepatitis A or typhoid. Table 2 shows that, 199 (65.3%) mobile food vendors and 128 (44.4%) of the canteen food vendors had poor knowledge of food safety. A higher proportion of the canteen food handlers 67 (23.3%) had good knowledge of food safety compared to mobile food vendors 22 (7.2%), p<0.01. The food safety and hygiene knowledge score for mobile and canteen food vendors ranged from 0 to 19 and 2 to 21, respectively with mean score ±SD of 8.0±3.8 and 9.0±3.6, respectively.

**Table 1:** socio-demographic characteristics of respondents

Socio-demographic characteristics	Mobile Food Vendors (n=305)	Canteen Food Vendors (n=288)		
Variables	n (%)	n (%)	χ^2^	p-value
Age (years)				
**≤20**	142 (46.6)	44 (15.2)	79.60	<0.001*
**21-30**	101 (33.0)	109 (37.9)		
**31-40**	42 (13.8)	82 (28.5)		
**41-50**	17 (5.6)	42 (14.6)		
**51-60**	3 (1.0)	11 (3.8)		
**Mean± SD**	24.6± 9.1	32.1± 10.3	t=12.10	P<0.001*
Sex				
**Male**	103 (33.8)	111 (38.5)	1.50	0.23
**Female**	202 (66.2)	177 (61.5)		
Ethnic group				
**Hausa**	216 (70.8)	191 (66.3)	12.30	0.02*
**Fulani**	53 (17.4)	36 (12.5)		
**Yoruba**	14 (4.6)	32 (11.1)		
**Igbo**	15 (4.9)	20 (7.0)		
**Others**	7 (2.3)	9 (3.1)		
Religion				
**Christianity**	29 (9.5)	44 (15.3)	4.60	0.03*
**Islam**	276(90.5)	244 (84.7)		
Educational status				
**None**	58 (19.0)	65 (22.5)	7.50	0.06
**Primary**	70 (23.0)	42 (14.6)		
**Secondary**	146 (48.0)	144 (50.0)		
**Tertiary**	31 (10.0)	37 (12.9)		
Marital status				
**Married**	63 (20.7)	92 (32.0)	39.40	<0.001*
**Single**	208 (68.0)	131 (45.5)		
**Divorced**	21 (7.0)	24 (8.3)		
**Widowed**	13 (4.3)	33 (11.4)		
**Separated**	0 (0.0)	8 (2.8)		
Duration in Business (years)				
**<5**	162 (53.0)	93 (32.3)	26.20	<0.001*
**≥5**	143 (47.0)	195 (67.7)		
Where food Preparation was learnt				
**Catering School**	28 (9.0)	45 (15.6)	11.80	0.008*
**Parents**	189 (62.0)	148 (51.4)		
**Friends**	34 (11.0)	49 (17.0)		
**Self**	54 (18)	46 (16.0)		
Hours of work(week)				
**<35**	133 (43.6)	125 (43.4)	0.0030	0.96
**≥35**	172 (56.4)	163 (56.6)		
Job Description				
**Food Preparation**	6 (2.0)	33 (11.5)	25.10	<0.001*
**Food Serving**	125 (41.0)	87 (30.2)		
**Food Preparation and Serving**	174 (57.0)	168 (58.3)		
Monthly income (Naira)				
**<18,000**	233 (73.1)	144 (50.0)	36.90	<0.001*
**≥18,000**	82 (26.9)	144 (50.0)		
Vaccinated against hepatitis/typhoid				
**Yes**	107 (35.0)	103 (35.8)	1.50	0.5
**No**	112 (37.0)	116 (40.2)		
**Don’t Know**	86 (28.0)	69 (24.0)		

* Statistically significant

**Table 2:** overall food safety knowledge among respondents

Overall food safety knowledge	Mobile Food Vendors (n=305)	Canteen Food Vendors (n=288)	χ^2^	p-value
	n (%)	n (%)		
Poor	199 (65.3)	128 (44.4)	38.20	<0.010*
Fair	84 (27.5)	93 (32.3)		
Good	22 (7.2)	67 (23.3)		
Mean	8.0	9.9		
Standard deviation	3.8	3.6		
Range	0.19	2.21		

* Statistically significant

Table 3 shows that for food preservation/storage, more canteen food vendors 233 (80.9%) than mobile food vendors 211 (69.2%) correctly answered the methods used in preparing vegetables before eating. Similarly, regarding circumstances that can lead to foodborne illnesses if practiced by food vendors, eating with unwashed hands after defecation and food contamination was correctly answered by more canteen food vendors 280 (97.2%) than mobile food vendors 250 (82.0%). Periodic medical examination was correctly answered by 145 (50.3%) canteen food vendors compared with 128 (42.0%) of mobile food vendor as one of the measures targeted to reduce foodborne illness. Wearing apron to prevent contamination and risk of infection was mentioned by 151 (52.4%) canteen food vendors compared with 108 (35.4%) mobile food vendors as the correct food safety measures used while serving food. For methods of food display, the use of covered containers to prevent dust and other possible contaminants was correctly mentioned by 166 (57.6%) canteen food vendors compared with 119 (39.0%) mobile food vendors. Table 4 showed that female mobile food vendors 17 (8.4%) had significantly higher proportion of good knowledge of food safety and hygiene compared with male food vendors 5 (4.9%), p=0.02; while male canteen food vendors 27 (24.3%) had significantly higher proportion of good knowledge of food safety and hygiene compared with female canteen food vendors 40 (22.6%), p=0.02. In addition, mobile food vendors 9 (10.5%), that do not know of their hepatitis/typhoid vaccination status had significantly higher proportion of good knowledge of food safety and hygiene compared with those who were aware of their vaccination status (4 (3.7%)), p=0.003; while canteen food vendors who were not vaccinated (42 (36.2%)) had significantly higher proportion of good knowledge of food safety and hygiene compared with those who were vaccinated (19 (18.4%), p<0.001).

**Table 3:** correct responses to parameters used to assess knowledge of food safety among respondents

Knowledge of food safety	Mobile food Vendors (n=305) n (%)	Canteen food Vendors (n=288) n (%)	χ^2^	p-value
Food preservation/storage				
Methods used in preparing vegetables before eating	211 (69.2)	233(80.9)	11	0.001*
Method used in treating raw food before cooking	190 (62.3)	185(64.2)	0.20	0.60
How the food vendors preserved fish/meat	164 (53.8)	177(61.5)	3.60	0.060
Handling of items that get spoilt during transportation	151 (49.5)	159(55.2)	2.0	0.20
Correct method of storing/preserving raw food	140 (45.9)	149(51.7)	2.0	0.20
Circumstances that can lead to food borne illnesses if practiced by food vendors				
Eating with unwashed hands after defecation and food contamination	250 (82.0)	280 (97.2)	36.30	<0.001*
Role of dirty environment and disease transmission	141 (46.2)	169 (58.7)	9.20	0.002*
Role of undercooked food in disease transmission	111 (36.4)	142 (49.3)	10.10	0.001*
Eating food that has stayed overnight unpreserved by the consumers	86 (28.2)	129 (44.8)	17.70	<0.001*
Role of chemical additives applied during storage	41 (13.4)	51 (17.7)	2.10	0.20
Which of the following reduces episodes of food borne illness				
Ensuring a good environmental sanitation	121 (39.7)	173 (60.1)	24.70	<0.001*
Keeping a good personal hygiene	130 (42.6)	167 (58.0)	14.0	<0.001*
Periodic medical examination	128 (42.0)	145 (50.3)	4.20	0.04*
Educating the public on causes of food borne illnesses	90 (29.5)	146 (50.7)	27.4	<0.001*
Washing raw food stuffs before cooking	90 (29.5)	99 (34.4)	1.60	0.2
Use of hand gloves, face mask and cap during food preparation	33 (10.8)	64 (22.2)	14.10	<0.001*
Food safety measures used while serving food				
Wearing apron prevent contamination and risk of infection	108 (35.4)	151 (52.4)	17.40	<0.001*
Wearing cap prevent contamination and risk of infection	86 (28.2)	110 (38.2)	6.70	0.01*
Wearing hand gloves prevent contamination and risk of infection	38 (12.8)	92 (31.9)	11.10	0.001*
Wearing face mask prevent contamination and risk of infection	24(7.9)	43 (14.9)	7.40	0.007*
Methods of food display				
Covered containers prevent dust and other possible contaminants	119 (39.0)	166 (57.6)	20.60	<0.001*
The use of show glass in protecting food is essential in preventing contamination	12 (3.9)	43 (14.9)	21.30	<0.001*
Fly proof nets is needed in preventing contamination of food by flies	9(3.0)	6 (2.1)	0.40	0.5†
Keeping prepared food opened can result in contamination	6 (2.0)	3 (1.0)		0.4†
None use of personal protective equipment by infected food handlers can contaminate food	4 (1.3)	3 (1.0)		0.8†

* Statistically significant, †=Fishers exact

**Table 4:** factors associated with food safety and hygiene knowledge among respondents

	Mobile Food Vendors Knowledge of Food Safety					Canteen Food Vendors Knowledge of Food Safety				
	Poor	Fair	Good	χ^2^	p-value	Poor	Fair	Good	χ^2^	p-value
**Age (years)**										
≤24	113 (61.4)	56 (30.4)	15 (8.2)	3.0	0.50	35 (49.3)	23 (32.4)	13 (18.3)	1.50	0.50
>24	86 (71.1)	28 (23.1)	7 (5.8)			93 (42.9)	70 (32.3)	54 (24.9)		
**Sex**										
Male	81 (78.6)	17 (16.5)	5 (4.9)	12.40	0.02*	61 (55.0)	23 (20.7)	27 (24.3)	12.10	0.020*
Female	118 (58.4)	67 (33.2)	17 (8.4)			67 (37.9)	70 (39.5)	40 (22.6)		
**Ethnic group**										
Hausa/Fulani	175 (65.1)	75 (27.9)	19 (7.1)	0.20	0.90	106 (46.7)	69 (30.4)	52 (22.9)	2.50	0.30
Others	24 (66.7)	9 (25.0)	3 (8.3)			22 (36.1)	24 (39.3)	15 (24.6)		
**Educational status**										
Primary	49 (70)	18 (25.7)	3 (4.3)			22 (52.4)	17 (40.5)	3 (7.1)		
Secondary	89 (61.0)	42 (28.8)	15 (10.3)	4.90	0.60	60 (41.7)	47 (32.6)	37 (25.7)	0.9	0.60
Tertiary	21 (67.7)	8 (25.8)	2 (6.5)			16 (43.2)	11 (29.7)	10 (27.0)		
Non	40 (69.0)	16 (27.6)	2 (3.4)			30 (46.2)	18 (27.7)	17 (26.2)		
**Marital status**										
Married	45 (71.4)	12(19.0)	6(9.5)	3.10	0.20	53 (57.6)	14 (15.2)	25 (27.2)	18.4	<0.010*
Unmarried	154 (63.6)	72(29.8)	16(6.6)			75 (38.3)	79 (40.3)	42 (21.4)		
**Duration in business (years)**										
<5	106 (65.4)	47(29.0)	9(5.6)	1.60	0.50	30 (32.3)	35 (37.6)	28 (30.1)	8.6	0.0140*
≥5	93 (65.0)	37(25.9)	13(9.1)			98 (50.3)	58 (29.7)	39 (20.0)		
**Learnt food preparation**										
Catering school	16 (57.1)	9 (32.1)	3 (10.7)			18 (40.0)	13 (28.9)	14 (31.1)	16	0.0140*
Parents	113 (59.8)	60 (31.7)	16 (8.5)			59 (39.9)	54 (36.5)	35 (23.6)		
Friends	25 (73.5)	8 (23.5)	1 (2.9)			19 (38.8)	19 (38.8)	11 (22.4)		
Self	45 (83.3)	7 (13.0)	2 (3.7)			32 (69.6)	7 (15.2)	7 (15.2)		
**Hours of work (week)**										
<35	90 (67.7)	38 (28.6)	5 (3.8)	4.20	0.10	73 (58.4)	33 (26.4)	19 (15.2)	18.2	<0.0010*
≥35	109 (63.4)	46 (26.7)	17 (9.9)			55 (33.7)	60 (36.8)	48 (29.4)		
Job Description										
Food Preparation	4 (66.7)	1 (16.7)	1 (16.7)			7 (21.2)	13 (39.4)	13 (39.4)	9.3	0.010*
Food Preparation &Serving	195 (65.2)	83 (27.8)	21 (7.0)			121 (47.5)	80 (31.4)	54 (21.2)		
**Monthly income(naira)**										
<18,000	155 (69.5)	55 (24.7)	13 (5.8)	7.0	0.030*	59 (41.0)	47 (32.6)	38 (26.4)	2	0.40
≥18,000	44 (53.7)	29 (35.4)	9 (11.0)			69 (47.9)	46 (41.9)	29 (20.1)		
**Vaccinated against Hepatitis/Typhoid**										
Yes	85 (79.4)	18 (16.8)	4 (3.7)	16.30	0.0030*	50 (48.5)	34 (33.0)	19 (18.4)	25.5	<0.0010*
No	62 (55.4)	41 (36.6)	9 (8.0)			38 (32.8)	36 (31.0)	42 (36.2)		
Don’t Know	52 (60.5)	25 (29.1)	9 (10.5)			40 (58.0)	23 (33.3)	6 (8.7)		

* Statistically significant

Using a logistic regression model, duration of work hours per week significantly predicted food safety knowledge among mobile food vendors. Mobile food vendors who worked for less than 35 hours per week were 70% less likely to have good knowledge of food safety [AOR=0.3, 95% CI= (0.2-0.6), p<0.001] relative to those who worked for ≥35 hours per week. Among canteen food handlers, married food vendors were 1.7 times more likely to have good knowledge of food safety and hygiene than unmarried food handlers [AOR=1.7, 95% CI= (1.2-2.3), p=0.002]. Similarly, canteen food handlers working ≥35 hours per week were 2.7 times more likely to have good knowledge of food safety and hygiene [AOR= 2.7, 95% CI= (1.6-4.3), p<0.001], while those involved in food preparation had 50% less likely to having good knowledge of food safety and hygiene [AOR= 0.5, 95%CI= (0.3-0.9), p=0.008], than those involved in combine food preparation and serving as shown in Table 5, Table 6.

**Table 5:** predictors of food safety and hygiene knowledge among street food vendors

	Predictors of street food vendors knowledge of food safety					
	**Poor**	**Fair**	**Good**	**COR**	**p-value**	**AOR (95%CI)**
**Sex**						
Male	78.6	16.5	4.9	0.3	0.3	1.3(0.8-2.3)
Female (Reference)	58.4	33.2	8.4			1
**Marital status**						
Married	71.4	19	9.5			
Unmarried (Reference)	63.6	29.8	6.6			1
**Duration in Business (years)**						
<5	65.4	29	5.6			
≥5 (Reference)	65	25.9	9.1			1
**Learnt Food Preparation**						
Catering School	57.1	32.1	10.7			
Parents	59.8	31.7	8.5			
Friends	73.5	23.5	2.9			
Self (Reference)	83.3	13	3.7			1
**Hours of work (week)**						
<35	67.7	28.6	3.8	-1.1	<0.001*	0.3(0.2-0.6)
≥35 (Reference)	63.4	26.7	9.9		1	
**Job Description**						
Food Preparation	66.7	16.7	16.7			
Food Preparation &Serving (Reference)	65.2	27.8	7			1
**Monthly income (Naira)**						
<18,000	69.5	24.7	5.8	0.5	0.1	1.7(0.9-3.2)
≥18,000 (Reference)	53.7	35.4	11			1
**Vaccinated against Hepatitis/Typhoid**						
Yes	79.4	16.8	3.7	0.2	0.3	1.2(0.9-1.6)
No	55.4	36.6	8			
Don’t Know (Reference)	60.5	29.1	10.5			1

Statistically significant, AOR=Adjusted odds ratio, CI= Confidence interval, Blank cells= Did not qualify for inclusion in the model

## Discussion

This study assessed and compared food safety knowledge among mobile food vendors and canteen food handlers in markets in Kano Metropolis, Northwest Nigeria. It is noteworthy that, good knowledge of food safety and hygiene is a key and an important intervention towards preventing the outbreak of foodborne diseases among the consumers, potentially because food handlers play a significant role in ensuring safety of food throughout the chain, from farm to plate [2], particularly because, a good number of households and traders often spend more time outside home as evidenced by a study that reported 67% of households to cook only once a day and often buy one to two meals of ready-to-eat food from food vendors [10].

Our study found out the proportion of mobile food vendors with good knowledge of food safety and hygiene to be 7.2%. However, a study conducted in Kano reported 62.4% of the mobile vendors interviewed to have good knowledge of food safety and hygiene [22]. This significant difference in terms of the level of knowledge food safety and hygiene is in keeping with the findings of 81% by a study conducted in South-Eastern Nigeria [26], and other studies conducted in Ghana [27,28] and Sri Lanka [29]. Even though all the studies were conducted among mobile food vendors, our study targeted mobile food vendors in the markets within the metropolis who are likely to be more concerned with fast food preparation to meet up with the demands of the busy traders within the markets. They are also unlikely to have a permanent vending site when compared with others who were studied at different locations outside the markets when compared with other studies highlighted above.

The World Health Organization reported that, about 1.8 million mortalities associated with diarrheal diseases in 2005, mainly due to the ingestion of contaminated food and drinking water [2,8]. Though this data may be under reported in developing countries including Nigeria, our finding of poor knowledge of food safety and hygiene among mobile food vendors is a pointer to an important risk factor of food contamination which can result in food borne illnesses and associated consequences. However, in the area of food preservation, our findings regarding the methods used in preparing vegetables before eating was 69.2%, which was better than 50.5% reported by a study conducted in Kano [22], perhaps due to the fact that vegetables are among the commonly consumed mobile vended food in the markets which may influence learning of how best it can be prepared. Similarly, regarding the circumstances that can lead to food borne illnesses if practiced by food vendors, eating with unwashed hands after defecation and food contamination, we found a lower positive response of 82% when compared with 95.8% by another study conducted in Kano [22]. This is not surprising, because hand washing is one of the important components of basic personal hygiene in Nigeria.

In the same vain, our study found 23.3% canteen food handlers to have good knowledge of food safety and hygiene, lower than the finding of 75.3% of canteen food handlers by a study conducted in Sokoto, Northwestern Nigeria [23], 68% by a study conducted in Oregon [30], 65% in Italy [31], 71% in Chicago and Neuchâtel, Switzerland respectively [32,33] and also 72% in Chicago [34]. The differences in food safety and hygiene knowledge compared with our findings may be related to the lack of training of canteen food handlers on food safety and hygiene and issuance of certificate to the food vendors before commencing food vending activities by the relevant agencies. It is noteworthy that food canteens were reported to be a potential source of a large number of foodborne outbreaks, as evidenced by 2007 report, which showed that, of the 1,097 outbreaks reported to the Centers for Disease Control and Prevention (CDC), 41% were associated with canteens [32]. The lower level reported by our study signifies the need for urgent intervention in line with food safety and hygiene guidelines to ensure that food vendors have the basic knowledge of food safety and hygiene, otherwise, the consumers will continue to be at increased risk of eating contaminated food and presumably higher incidence of food borne diseases and the associated complications. It is therefore of paramount importance if the agencies responsible for certifying food vendors can put in place preconditions for certification and re-certification of canteen food handlers, for example a mandatory certificate of training on food safety and hygiene by a registered catering school.

Furthermore, when compared with the above studies, our finding found significantly lower food safety and hygiene knowledge among the two categories of food vendors, particularly among the mobile food vendors, posing a significant risk of food borne diseases among consumers, perhaps due to the fact that the vendors do not have permanent vending sites, which if available, can serve as a platform by relevant stakeholders for training, retraining and supervision, to ensure that all the vendors have acquired the basic knowledge and skills in the areas of food safety and hygiene. It is therefore pertinent that, all the stakeholders responsible for ensuring food safety and hygiene in Kano should ensure a formidable and sustained collaboration targeted towards registration, supervision and perhaps fixed vending site for mobile food vendors. This can go a long way in providing accountable platform for improving knowledge of food safety and hygiene. Overall, having good knowledge of food safety and hygiene among food vendors is critical in preventing food borne diseases among consumers. This is so because, knowledgeable food vendors are more likely to comply with recommended guidelines targeted towards ensuring safety of the vended foods. Therefore, the results of our study highlighted the need for sustained efforts in enforcing the food safety and hygiene guidelines.

Our study reported better response among mobile food vendors of 82% and canteen food handlers of 97% regarding hand washing after going to the toilet respectively, but lower values regarding the use of gloves among the mobile food vendors of 10.8% and canteen food handlers of 22.2%, we also found out that, some food vendors, especially the mobile food vendors, were not aware of the role of periodic medical examination in the prevention of food contamination, despite the existing evidence of the role of periodic medical examination in the prevention of food contamination by food handlers [17]. These findings signifies the need for improving the other areas of preventing food contamination among food vendors in Kano like periodic medical examination, while sustaining and improving the areas of strengths, like personal hygiene and environmental sanitation and emphasizing the role of contaminated fingers, flies, faeces, fluid and food in the transmission of food borne diseases, [2,3,17] and that, prevention of food contamination requires knowledge around eliminating the risks of food contamination.

**Limitations:** this study was limited by social desirability bias which was reduced by proper training of the research assistants on how to minimize the bias through good communication to the eligible respondents on the importance of providing correct information that can be of help in policy formulation and regular training of food vendors.

## Conclusion

Food safety and hygiene knowledge was found to be suboptimal among the two groups of food vendors and this potentially exposes the consumers to the high risk of foodborne diseases which has negative socio-economic consequences to the affected consumers, the families, communities, health system and the country at large. Recommendations Government should ensure enforcement of the existing policy targeting food safety and hygiene among food vendors. Similarly, regular training should be ensured by appropriate government agency.

### What is known about this topic

- *Poor knowledge of food safety and hygiene was reported by various studies particularly among street vendors compared to the canteen food vendors;*
- *Canteen food handlers have permanent vending sites, and are more likely to be trained and regularly supervise as such they have better knowledge of food safety and hygiene.*

### What this study adds

- *The study provided a comparative data on food safety and hygiene among the two groups of food vendors which could be of use by policy makers in designing strategies for training, supervision and enforcement of food safety and hygiene guidelines.*
